# Association between fatigue and functional status among patients with multiple sclerosis

**DOI:** 10.3389/fneur.2026.1917177

**Published:** 2026-08-05

**Authors:** Esraa Ezzat Mohamed Abo Zaid, Eman Talaat Mohammed Elshamaa, Arzak Mohamed Khalifa

**Affiliations:** Department of Medical Surgical Nursing, Faculty of Nursing, Ain Shams University, Cairo, Egypt

**Keywords:** activities of daily living, fatigue, functional status, multiple sclerosis, walking impairment

## Abstract

**Background:**

Fatigue is one of the most common and disabling symptoms experienced by patients with multiple sclerosis (MS). This study aimed to assess the association between fatigue and functional status among patients with multiple sclerosis.

**Methods:**

A descriptive correlational cross-sectional study was conducted in September 2025 to January 2026, on 78 patients diagnosed with MS using a purposive sampling method, at Nasser Institute Hospital, Egypt. Data were collected using a structured interview questionnaire, the Fatigue Severity Scale, Lawton and Brody’s Instrumental Activities of Daily Living Scale (IADL), the MS Walking Scale, and the Social Functioning Questionnaire. Data were analyzed using SPSS version 25. Statistical significance was considered at *p* < 0.05.

**Results:**

After assessment using the Fatigue Severity Scale, 46.1% of participants were classified as having severe fatigue, 52.6% had moderate fatigue, and 1.3% had mild fatigue. Female participants reported higher fatigue scores than males. More than half of the participants demonstrated impaired social functioning, and a considerable proportion exhibited dependence in instrumental activities of daily living and moderate-to-severe walking impairment. Significant positive correlations were observed between fatigue severity and dependence in instrumental activities of daily living, walking impairment, and social dysfunction (all *p* < 0.001). In the multiple linear regression analysis, after adjusting for confounding variables, the overall model was statistically significant (*R*^2^ = 0.545, adjusted *R*^2^ = 0.500, *F* = 11.984, *p* < 0.001). Greater walking impairment (MSWS-12; *β* = 0.371, *p* = 0.010) and poorer instrumental activities of daily living (IADL; *β* = 0.311, *p* = 0.033) remained independently associated with higher fatigue severity, whereas social functioning was not independently associated with fatigue severity after adjustment (*β* = 0.200, *p* = 0.095).

**Conclusion:**

Fatigue is highly prevalent among patients with MS and is significantly associated with poorer functional status, particularly reduced instrumental activities of daily living and greater walking impairment. Routine assessment of fatigue and the development of comprehensive fatigue-management strategies may help identify patients at risk of functional limitations and support the delivery of individualized care aimed at maintaining independence, mobility, and quality of life among individuals with MS.

## Introduction

Multiple sclerosis (MS) is a chronic immune-mediated inflammatory and neurodegenerative disease of the central nervous system and one of the leading causes of non-traumatic disability among young adults worldwide (1, 2). The disease is characterized by a wide range of motor, sensory, cognitive, and psychological manifestations that substantially impair daily functioning, social participation, and quality of life (3). Although disease-modifying therapies have improved clinical outcomes, many individuals with MS continue to experience disabling symptoms that adversely affect long-term functional independence (4).

Fatigue is among the most prevalent and disabling symptoms of MS, affecting approximately 59–90% of patients across different stages of the disease (5, 6). A recent systematic review and meta-analysis estimated the global prevalence of MS-related fatigue at 59.1%, underscoring its substantial clinical and public health burden (7). Unlike normal tiredness, MS-related fatigue is characterized by persistent physical and cognitive exhaustion that is disproportionate to exertion and inadequately relieved by rest (8). Fatigue has been consistently associated with reduced physical activity, impaired mobility, decreased work productivity, and poorer quality of life (5, 9). Previous studies have also demonstrated that fatigue may interfere with instrumental activities of daily living, contribute to gait and balance impairment, and limit social participation, thereby increasing functional dependence among individuals with MS (10, 11). Despite these findings, fatigue remains one of the most challenging symptoms to assess and manage in routine clinical practice because of its multifactorial nature and complex interaction with physical, psychological, and disease-related factors (6).

Functional status is a key outcome in the management of MS because it reflects an individual’s ability to maintain independence and fulfill physical, social, and occupational roles (12). Preserving functional ability is a major goal of rehabilitation and nursing care, as functional decline is associated with increased healthcare utilization, reduced quality of life, and greater caregiver burden (13). Consequently, identifying modifiable factors that contribute to functional impairment is essential for developing effective patient-centered interventions. Although numerous international studies have examined fatigue in MS, most have focused on overall disability, quality of life, cognitive impairment, sleep disturbances, or disease progression rather than comprehensively evaluating multiple domains of functional status (14–16). Relatively few studies have simultaneously investigated the relationship between fatigue and instrumental activities of daily living, walking ability, and social functioning within the same patient population. Furthermore, evidence from Egypt and other Middle Eastern countries remains limited, restricting the availability of context-specific data to guide clinical practice and rehabilitation planning. This knowledge gap limits healthcare professionals’ ability to identify patients at greatest risk of functional decline and to implement targeted interventions that address the diverse consequences of fatigue.

By simultaneously examining instrumental activities of daily living, walking impairment, and social functioning, the present study provides a comprehensive assessment of the functional impact of fatigue among patients with MS. A better understanding of these relationships may facilitate early identification of patients at risk for disability, support individualized rehabilitation and nursing care, and ultimately improve functional independence and quality of life. Therefore, this study aimed to assess the association between fatigue and functional status among patients with multiple sclerosis.

## Materials and methods

### Study design

A descriptive cross-sectional study design was employed to examine the association between fatigue and functional status among patients with MS.

### Study setting and period

The study was conducted at the MS Day Care Unit, Neurology Department of Nasser Institute Hospital, Cairo, Egypt. Nasser Institute Hospital is a tertiary healthcare facility affiliated with the Egyptian Ministry of Health and provides specialized neurological services, including the diagnosis, treatment, and follow-up of patients with MS. The MS Day Care Unit operates 6 days per week, from Saturday to Thursday, between 9:00 a.m. and 2:00 p.m. On average, 5 to 7 patients with MS attend the unit daily for assessment, treatment, and follow-up care. The unit consists of a dedicated patient care room equipped with six treatment chairs and monitoring equipment and is staffed by three nurses specialized in patient care. Data collection was carried out over a four-month period, commencing in September 2025 and continuing through January 2026.

### Study population

The study population consisted of adult patients diagnosed with MS who attended the MS Day Care Unit at Nasser Institute Hospital, Cairo, Egypt, during the study period. Patients were recruited from those attending the unit for routine follow-up, assessment, and treatment. Participants represented both sexes and different clinical characteristics of MS.

### Eligibility criteria

Patients were eligible to participate in the study if they were adults aged 18 years or older, had a confirmed diagnosis of multiple sclerosis established by a neurologist according to standard diagnostic criteria, were clinically stable at the time of data collection, attended the MS Day Care Unit for routine follow-up or treatment, were able to communicate verbally and understand the study questionnaire, and provided written informed consent. Fatigue severity was subsequently assessed in all enrolled participants using the Fatigue Severity Scale. Patients were excluded if they were experiencing an acute relapse or exacerbation of MS during the data collection period, as this could affect fatigue levels and functional status. Patients with chronic musculoskeletal disorders, such as osteoarthritis or other mobility-limiting conditions, were also excluded to avoid confounding the assessment of functional status and walking ability. Furthermore, patients with severe mental or psychological disorders, cognitive impairment that interfered with understanding or responding to the questionnaire, other neurological diseases affecting physical function, or severe medical conditions that could independently contribute to fatigue or disability were not eligible for inclusion in the study.

### Sample size and sampling technique

The sample size was determined based on the total number of patients with MS attending the study setting during 2024 (*N* = 400) (hospital archive). Using Thompson’s formula for finite populations, with a confidence level of 95% (*Z* = 1.96), a margin of error of 10% (*d* = 0.10), and an estimated population proportion of 50% (*p* = 0.50), the minimum required sample size was calculated to be 78 participants (17). A purposive sampling technique was employed to recruit eligible participants. Patients attending the MS Day Care Unit during the data collection period were screened according to the predefined eligibility criteria, and those who met the inclusion criteria and agreed to participate were consecutively enrolled until the required sample size of 78 patients was achieved.

### Data collection

Data were collected through face-to-face interviews using a structured questionnaire composed of six parts. Part 1: Sociodemographic characteristics. This researcher-developed section was constructed following an extensive review of the literature (18–21) and included age, sex, marital status, educational level, and other relevant variables. Part 2: Clinical characteristics. This section assessed weight, height, body mass index (BMI), disease subtype, age at symptom onset, relapse frequency, and other disease-related variables. Part 3: Fatigue severity scale (FSS). The standard Fatigue Severity Scale (FSS) is a 9-item questionnaire. While the original and most commonly validated format uses a 7-point Likert scale (1 = strongly disagree to 7 = strongly agree). In the present study, a modified three-point response format (1 = disagree, 2 = neutral/sometimes, and 3 = agree) was used to simplify administration in the study setting (18). In the current study, the FSS was administered as a nine-item instrument using a three-point Likert scale (1 = disagree to 3 = agree), with total scores ranging from 9 to 27. Higher scores indicate greater fatigue severity. Fatigue was classified as mild (≤15), moderate (16–21), and severe (22–27) according to the criteria reported by Vold et al. (18). Part 4: Functional status: Lawton and Brody Instrumental Activities of Daily Living Scale (IADL). This scale assesses independence in eight domains: telephone use, shopping, food preparation, housekeeping, laundry, transportation, medication management, and financial management. Each item is scored as 0 (dependent) or 1 (independent), yielding a total score of 0–8, with higher scores indicating greater independence (19). Part 5: Walking impairment: The updated version of the Multiple Sclerosis Walking Scale (MSWS-12v2) was used. In the present study, a modified three-point response format (1 = not at all, 2 = moderately, and 3 = extremely) was used to facilitate questionnaire administration. This 12-item scale is rated on a three-point scale and assesses walking-related difficulties over the previous 2 weeks. Higher scores indicate greater walking impairment according to the criteria reported by Dib et al. (20). And part 6: Social functioning: Social Functioning Questionnaire (SFQ). This eight-item scale assesses social, occupational, family, and leisure functioning. Total scores are summed, with scores <10 indicating normal social functioning and scores ≥10 indicating impaired social functioning (21).

### Validity and reliability

Content validity was assessed only for the researcher-developed questionnaire, which included the sociodemographic and clinical characteristics sections (Parts 1 and 2). These sections were reviewed by a panel of experts in medical-surgical nursing, neurology, and research methodology to evaluate the relevance, clarity, comprehensiveness, and appropriateness of the items for the study objectives. The experts’ comments were incorporated into the final version of the questionnaire. The Fatigue Severity Scale (FSS), Lawton and Brody Instrumental Activities of Daily Living Scale (IADL), Multiple Sclerosis Walking Scale (MSWS-12), and Social Functioning Questionnaire (SFQ) are standardized instruments with established validity and reliability that have been widely used in previous studies, including studies involving patients with multiple sclerosis.

To evaluate the performance of these instruments in the present study population, internal consistency reliability was assessed using Cronbach’s alpha coefficient. The FSS demonstrated good internal consistency (Cronbach’s *α* = 0.83), the IADL Scale showed good internal consistency (Cronbach’s *α* = 0.83), the MSWS-12 demonstrated excellent internal consistency (Cronbach’s α = 0.89), and the SFQ demonstrated good internal consistency (Cronbach’s α = 0.87). These findings indicate that the standardized instruments exhibited acceptable to excellent reliability in the current study sample.

### Pilot study

A pilot study was conducted on 10% of the calculated sample (*n* = 8) before the commencement of the main study to assess the feasibility, clarity, applicability, and administration procedures of the data collection instruments and to estimate the time required for questionnaire completion. No modifications to the study instruments were required based on the pilot findings. Although inclusion of pilot participants in the final analysis may be acceptable when no instrument modifications are made, the pilot participants were intentionally excluded because the pilot was conducted as a separate preliminary phase to evaluate study procedures rather than to contribute to hypothesis testing. Excluding these participants ensured methodological independence between the pilot and the main study and avoided any potential influence of prior exposure to the study procedures on the final results.

### Ethical considerations

Ethical approval was obtained from the Ethical Committee of the Faculty of Nursing, Ain Shams University (Approval No. 26.01.1100). Participants were informed about the study objectives and provided written informed consent prior to participation. Participation was voluntary, and participants had the right to withdraw at any time. Anonymity and confidentiality were ensured, and all collected data were used solely for research purposes.

### Statistical analysis

Data were entered, coded, and analyzed using the Statistical Package for the Social Sciences (SPSS) version 25. Descriptive statistics were used to summarize participants’ sociodemographic, medical, and clinical characteristics. Continuous variables were presented as means and standard deviations (Mean ± SD), while categorical variables were expressed as frequencies and percentages. Comparisons between male and female participants were performed using the independent samples t-test for continuous variables and categorical variables were compared using the chi-square test. When the assumptions of the chi-square test were violated (i.e., when more than 20% of the expected cell counts were less than 5 or any expected cell count was less than 1), Fisher’s exact test was used instead. Before conducting inferential analyses, the assumptions underlying the statistical tests were evaluated. The normality of continuous variables was assessed using the Shapiro–Wilk test and visual inspection of histograms and Q–Q plots. Homogeneity of variances for independent-samples *t*-tests was assessed using Levene’s test. For linear regression analyses, the assumptions of linearity, independence of errors, homoscedasticity, normality of residuals, and absence of multicollinearity were evaluated using scatterplots, standardized residual plots, normal probability (P–P) plots, the Durbin–Watson statistic, and variance inflation factors (VIFs), respectively. All assumptions were considered adequately satisfied before the analyses were performed. To examine the relationship between fatigue severity and functional outcomes, multiple linear regression analysis was conducted. Regression coefficients (B), standardized beta coefficients (*β*), standard errors (SE), *t*-values, 95% confidence intervals (95% CI), *R*^2^, adjusted *R*^2^, *F* values and *p*-values were reported. Statistical significance was considered at *p* < 0.05 for all analyses.

## Results

Table 1 presents the sociodemographic characteristics of the 78 study participants according to sex. The mean age of participants was 36.4 ± 8.4 years, with no significant difference between males and females (37.7 ± 8.8 vs. 36.2 ± 8.3 years, *p* = 0.550). Most participants were aged 30 to <40 years (38.5%), married (89.7%), and had a diploma or university education (60.3%), with no statistically significant sex differences in age category (*p* = 0.439), marital status (*p* = 0.601), or educational level (*p* = 0.604). A significantly higher proportion of males were employed compared with females (76.9% vs. 23.1%, *p* = 0.001). Likewise, the nature of work differed significantly by sex (*p* = 0.001). No statistically significant differences were observed between males and females regarding residence (*p* = 0.320), sufficiency of monthly income for treatment costs (*p* = 0.384), financial burden on the family due to illness and treatment costs (*p* = 0.454), mean family size (*p* = 0.791), or family size categories (*p* = 0.414).

**Table 1:** Sociodemographic characteristics of the study participants by sex.

Variables	Male *n* = 13 (16.7%)	Female *n* = 65 (83.3%)	Total *n* = 78 (100%)	*p* value
Age (years)				
Mean ± SD	37.7 ± 8.8	36.2 ± 8.3	36.4 ± 8.4	0.550
Age categories				
18 to <30 years	3.0 (23.1)	18 (27.7)	21 (26.9)	0.439
30 to <40 years	7.0 (53.8)	23 (35.4)	30 (38.5)	
40–60 years	3.0 (23.1)	24 (36.9)	27 (34.6)	
Marital status				
Single	1.0 (7.7)	7.0 (10.8)	8.0 (10.3)	0.601
Married	12 (92.3)	58 (89.2)	70 (89.7)	
Educational level				
Illiterate	1.0 (7.7)	3.0 (4.6)	4.0 (5.1)	0.604
Primary	0.0 (0.0)	7.0 (10.8)	7.0 (9.0)	
Secondary	3.0 (23.1)	17 (26.2)	20 (25.6)	
Diploma/University	9.0 (69.2)	38 (58.5)	47 (60.3)	
Working status				
Employed	10 (76.9)	15 (23.1)	25 (32.1)	0.001
Not employed	3.0 (23.1)	50 (76.9)	53 (67.9)	
If working, the nature of the work requires				
Muscular effort	6.0 (46.1)	9.0 (13.8)	15 (19.3)	0.001
Mental effort	4.0 (30.8)	6.0 (9.2)	10 (12.8)	
Both of them	3.0 (23.1)	5.0 (7.7)	8.0 (10.2)	
Not employed	3.0 (23.1)	50 (76.9)	53 (67.9)	
Residence				
Rural	5.0 (38.5)	18 (27.7)	23 (29.5)	0.320
Urban	8.0 (61.5)	47 (72.3)	55 (70.5)	
Monthly income sufficient for treatment costs				
Yes	4.0 (30.8)	26 (40.0)	30 (38.5)	0.384
No	9.0 (69.2)	39 (60.0)	48 (61.5)	
Is there a financial burden on family due to your illness and the costs of your treatment?				
Yes	7.0 (53.8)	39 (60.0)	46 (59.0)	0.454
No	6.0 (46.2)	26 (40.0)	32 (41.0)	
Family size				
Mean ± SD	1.92 ± 0.4	1.97 ± 0.5	1.96 ± 0.5	0.791
Family size categories				
1–2 individuals	12 (92.3)	55 (84.6)	67 (85.9)	0.414
3–5 individuals	1.0 (7.7)	10 (15.4)	11 (14.1)	

Categorical variables are presented as frequencies and percentages. Comparisons were performed using the chi-square test or Fisher’s exact test, as appropriate. Continuous variables were compared using the independent-samples *t*-test.

Table 2 summarizes the medical and clinical characteristics of the study participants by sex. Male participants had significantly higher mean weight (82.3 ± 17.8 vs. 71.5 ± 9.6 kg, *p* = 0.002), height (176.0 ± 7.8 vs. 163.1 ± 5.2 cm, *p* = 0.001), and body mass index (34.8 ± 9.6 vs. 26.9 ± 3.0 kg/m^2^, *p* = 0.001) than female participants. Relapsing–remitting MS was the most common disease subtype (70.5%), and most participants had experienced symptom onset more than 5 years previously (61.5%), with no significant sex differences in disease type (*p* = 0.742), duration since symptom onset (*p* = 0.746), or relapse frequency (*p* = 0.124). The severity of pain differed significantly between males and females (*p* = 0.020), with females reporting higher proportions of moderate and severe pain, whereas males more frequently reported mild or no pain. A significantly higher proportion of females had previously undergone surgery (69.2% vs. 30.8%, *p* = 0.011), while smoking was reported exclusively among male participants (61.5% vs. 0%, *p* = 0.001). No statistically significant sex differences were observed regarding regular neurologist follow-up (*p* = 0.448), presence of other chronic diseases (*p* = 0.471), previous hospitalization (*p* = 0.535), allergies (*p* = 0.609), family history of MS (*p* = 0.332), or degree of affected relatives (*p* = 0.809).

**Table 2:** Medical and clinical characteristics of the study participants by sex.

Variables	Male *n* = 13 (16.7%)	Female *n* = 65 (83.3%)	Total *n* = 78 (100%)	*p* value
Weight (kg)				
Mean ± SD	82.3 ± 17.8	71.5 ± 9.6	73.3 ± 11.9	0.002
Height (cm)				
Mean ± SD	176.0 ± 7.8	163.1 ± 5.2	165.3 ± 7.4	0.001
Body Mass Index (kg/m^2^)				
Mean ± SD	34.8 ± 9.6	26.9 ± 3.0	28.2 ± 5.6	0.001
What type of multiple sclerosis do you suffer from?				
Primary progressive multiple sclerosis	1.0 (7.7)	3.0 (4.6)	4.0 (5.1)	0.742
Secondary progressive multiple sclerosis	1.0 (7.7)	13 (20.0)	14 (17.9)	
Progressive relapsing multiple sclerosis	1.0 (7.7)	4.0 (6.2)	5.0 (6.4)	
Relapsing–remitting multiple sclerosis	10 (76.9)	45 (69.2)	55 (70.5)	
Onset of the first symptoms of multiple sclerosis				
Less than 1 year	2.0 (15.4)	6.0 (9.2)	8.0 (10.3)	0.746
1–5 years	4.0 (30.8)	18 (27.7)	22 (28.2)	
More than 5 years	7.0 (53.8)	41 (63.1)	48 (61.5)	
How many times have you had a relapse? (Frequency of relapse)				
1–5 times	9.0 (69.2)	26 (40.0)	35 (44.9)	0.124
6–10 times	4.0 (30.8)	33 (50.8)	37 (47.4)	
More than 10 times	0.0 (0.0)	6.0 (9.2)	6.0 (7.7)	
What is the severity of pain?				
No pain	3.0 (23.1)	6.0 (9.2)	9.0 (11.5)	0.020
Mild pain	5.0 (38.5)	7.0 (10.8)	12 (15.4)	
Moderate pain	3.0 (23.1)	27 (41.5)	30 (38.5)	
Severe pain	2.0 (15.4)	25 (38.5)	27 (34.6)	
Do you follow up regularly with a neurologist?				
Yes	5.0 (38.5)	21 (32.3)	26 (33.3)	0.448
No	8.0 (61.5)	44 (67.7)	52 (66.7)	
Do you suffer from other chronic diseases?				
Yes	2.0 (15.4)	14 (21.5)	16 (20.5)	0.471
No	11 (84.6)	51 (78.5)	62 (79.5)	
Have you been hospitalized before for another illness?				
Yes	1.0 (7.7)	8.0 (12.3)	9.0 (11.5)	0.535
No	12 (92.3)	57 (87.7)	69 (88.5)	
Have you previously had surgeries?				
Yes	4.0 (30.8)	45 (69.2)	49 (62.8)	0.011
No	9.0 (69.2)	20 (30.8)	29 (37.2)	
Do you suffer from allergies?				
Yes	1.0 (7.7)	4.0 (6.2)	5.0 (6.4)	0.609
No	12 (92.3)	61 (93.8)	73 (93.6)	
Does any member in your family have multiple sclerosis?				
Yes	2.0 (15.4)	17 (26.2)	19 (24.4)	0.332
No	11 (84.6)	48 (73.8)	59 (75.6)	
If the answer is yes, what is the degree of relationship?				
No	11 (84.6)	48 (73.8)	59 (75.6)	0.809
First degree	2.0 (15.4)	14 (21.5)	16 (20.5)	
Second degree	0.0 (0.0)	2.0 (3.1)	2.0 (2.6)	
Third degree	0.0 (0.0)	1.0 (1.5)	1.0 (1.3)	
Do you smoke?				
Yes	8.0 (61.5)	0.0 (0.0)	8.0 (10.3)	0.001
No	5.0 (38.5)	65 (100.0)	70 (89.7)	

Categorical variables are presented as frequencies and percentages. Comparisons were performed using the chi-square test or Fisher’s exact test, as appropriate. Continuous variables were compared using the independent-samples *t*-test.

Table 3 compares fatigue severity scale scores between male and female participants. Female participants demonstrated higher mean scores than males on nearly all fatigue severity scale items and on the total fatigue score. Nevertheless, most of these differences were not statistically significant (all *p*-values > 0.05). A significant sex-related difference was identified only for the item “Fatigue is among my three most disabling symptoms,” with females reporting significantly higher scores than males (2.46 ± 0.6 versus 1.85 ± 0.8, *p* = 0.003). Although female participants also exhibited higher mean scores for reduced motivation due to fatigue (*p* = 0.094), feeling easily fatigued (*p* = 0.054), experiencing frequent fatigue-related problems (*p* = 0.061), fatigue interference with work, family, or social activities (*p* = 0.094), and the overall fatigue severity score (*p* = 0.096), none of these differences achieved statistical significance. These results indicate a tendency toward greater fatigue severity among females; however, sex was not significantly associated with overall fatigue severity, except in relation to perceiving fatigue as one of the most disabling symptoms. Furthermore, analysis of fatigue severity categories showed that moderate fatigue was more common among males (69.2%) than females (49.2%), whereas severe fatigue was more prevalent among females (49.2%) compared with males (30.8%). Despite these differences, the distribution of fatigue severity categories did not vary significantly according to sex (*p* = 0.402).

**Table 3:** Fatigue severity scale scores of the study participants by sex.

Variables	Male *n* = 13 (16.7%)	Female *n* = 65 (83.3%)	Total *n* = 78 (100%)	*p* value
1. My motivation is lower when I am fatigued				
Mean ± SD	2.15 ± 0.8	2.48 ± 0.5	2.42 ± 0.6	0.094
2. Exercise brings on my fatigue				
Mean ± SD	2.38 ± 0.6	2.52 ± 0.5	2.50 ± 0.5	0.392
3. I am easily fatigued				
Mean ± SD	2.23 ± 0.5	2.55 ± 0.5	2.50 ± 0.5	0.054
4. Fatigue interferes with my physical functioning				
Mean ± SD	2.46 ± 0.5	2.48 ± 0.5	2.47 ± 0.5	0.930
5. Fatigue causes frequent problems for me				
Mean ± SD	2.08 ± 0.7	2.45 ± 0.6	2.38 ± 0.6	0.061
6. My fatigue prevents sustained physical functioning				
Mean ± SD	2.38 ± 0.5	2.54 ± 0.5	2.51 ± 0.5	0.317
7. Fatigue interferes with carrying out certain duties and responsibilities				
Mean ± SD	2.38 ± 0.6	2.51 ± 0.5	2.49 ± 0.5	0.447
8. Fatigue is among my three most disabling symptoms				
Mean ± SD	1.85 ± 0.8	2.46 ± 0.6	2.36 ± 0.7	0.003
9. Fatigue interferes with my work, family, or social life				
Mean ± SD	2.08 ± 0.7	2.42 ± 0.6	2.36 ± 0.6	0.094
Fatigue severity scale total scores				
Mean ± SD	2.22 ± 0.5	2.48 ± 0.5	2.44 ± 0.5	0.096
Fatigue severity categories				
Mild	0.0 (0.0)	1.0 (1.5)	1.0 (1.3)	0.402
Moderate	9.0 (69.2)	32 (49.2)	41 (52.6)	
Sever	4.0 (30.8)	32 (49.2)	36 (46.1)	

Categorical variables are presented as frequencies and percentages. Comparisons were performed using the chi-square test or Fisher’s exact test, as appropriate. Continuous variables were compared using the independent-samples *t*-test.

Table 4 presents the functional status of the study participants according to sex. Regarding instrumental activities of daily living (IADL), all participants were independent in their ability to use the telephone. Dependence was most common for shopping (84.6%) and food preparation (73.1%), whereas most participants remained independent in housekeeping (80.8%), laundry (56.4%), transportation (51.3%), medication management (82.1%), and financial management (83.3%). Male participants demonstrated significantly greater independence in shopping than female participants (46.2% vs. 9.2%, *p* = 0.004), and the overall IADL score indicated a significantly higher proportion of independent males compared with females (76.9% vs. 60.0%, *p* = 0.031). With respect to walking function, moderate walking impairment was the most common category (52.6%), followed by severe impairment (29.5%) and no impairment (17.9%). Female participants reported significantly greater need for indoor walking support than males (mean ± SD: 1.92 ± 0.7 vs. 1.31 ± 0.6, *p* = 0.009), and the distribution of overall walking impairment categories differed significantly between the sexes (*p* = 0.009). No other individual walking items showed statistically significant sex differences. Regarding social functioning, 59.0% of participants exhibited significant social impairment. Female participants reported significantly greater difficulty completing work and home tasks (1.94 ± 0.8 vs. 1.31 ± 0.9, *p* = 0.015) and significantly higher stress related to these responsibilities (2.34 ± 0.7 vs. 1.46 ± 0.9, *p* = 0.001) than males. No statistically significant sex differences were observed for the remaining social functioning domains or for the overall social functioning categories (*p* = 0.762).

**Table 4:** Functional status of the study participants by sex.

Variables	Male *n* = 13 (16.7%)	Female *n* = 65 (83.3%)	Total *n* = 78 (100%)	*p* value
PART I: The Lawton Instrumental Activities of Daily Living (IADL) Scale				
1. Ability to use telephone				
Dependent (0)	0.0 (0.0)	0.0 (0.0)	0.0 (0.0)	–
Independence (1)	13 (100.0)	65 (100.0)	78 (100)	
2. Shopping				
Dependent (0)	7.0 (53.8)	59 (90.8)	66 (84.6)	0.004
Independence (1)	6.0 (46.2)	6.0 (9.2)	12 (15.4)	
3. Food preparation				
Dependent (0)	7.0 (53.8)	50 (76.9)	57 (73.1)	0.089
Independence (1)	6.0 (46.2)	15 (23.1)	21 (26.9)	
4. Housekeeping				
Dependent (0)	5.0 (38.5)	10 (15.4)	15 (19.2)	0.068
Independence (1)	8.0 (61.5)	55 (84.6)	63 (80.8)	
5. Laundry				
Dependent (0)	3.0 (23.1)	31 (47.7)	34 (43.3)	0.132
Independence (1)	10 (76.9)	34 (52.3)	44 (56.4)	
6. Mode of transportation				
Dependent (0)	4.0 (30.8)	34 (52.3)	38 (48.7)	0.226
Independence (1)	9.0 (69.2)	31 (47.7)	40 (51.3)	
7. Responsibility for own medications				
Dependent (0)	1.0 (7.7)	13 (20.0)	14 (17.9)	0.443
Independence (1)	12 (92.3)	52 (80.0)	64 (82.1)	
8. Ability to handle finances				
Dependent (0)	1.0 (7.7)	12 (18.5)	13 (16.7)	0.683
Independence (1)	12 (92.3)	53 (81.5)	65 (83.3)	
IADL scale total scores categories				
Dependent (0)	3.0 (23.1)	26 (40.0)	29 (37.2)	0.031
Independence (1)	10 (76.9)	39 (60.0)	49 (62.8)	
Part II: Multiple Sclerosis Walking Scale (MSWS): In the past 2 weeks, how much has your MS?				
1. Limited your ability to walk?				
Mean ± SD	2.08 ± 0.8	2.20 ± 0.6	2.18 ± 0.6	0.565
2. Limited your ability to run?				
Mean ± SD	2.31 ± 0.8	2.60 ± 0.6	2.55 ± 0.6	0.145
3. Limited your ability to climb up and down stairs?				
Mean ± SD	2.15 ± 0.8	2.45 ± 0.6	2.40 ± 0.6	0.153
4. Made standing when doing things more difficult?				
Mean ± SD	2.08 ± 0.9	2.15 ± 0.6	2.14 ± 0.7	0.732
5. Limited your balance when standing or walking?				
Mean ± SD	2.08 ± 0.8	2.40 ± 0.6	2.35 ± 0.6	0.108
6. Limited how far you are able to walk?				
Mean ± SD	1.92 ± 0.9	2.25 ± 0.6	2.19 ± 0.6	0.121
7. Increased the effort needed for you to walk?				
Mean ± SD	1.92 ± 0.9	2.23 ± 0.5	2.18 ± 0.6	0.125
8. Made it necessary for you to use support when walking indoors (e.g., holding on to furniture, using a stick, etc.)?				
Mean ± SD	1.31 ± 0.6	1.92 ± 0.7	1.82 ± 0.7	0.009
9. Made it necessary for you to use support when walking outdoors (e.g., using a stick, a frame, etc.)?				
Mean ± SD	1.46 ± 0.8	1.89 ± 0.7	1.82 ± 0.7	0.059
10. Slowed down your walking?				
Mean ± SD	1.85 ± 0.8	2.17 ± 0.5	2.12 ± 0.6	0.099
11. Affected how smoothly you walk?				
Mean ± SD	1.85 ± 0.8	2.12 ± 0.6	2.08 ± 0.6	0.169
12. Made you concentrate on your walking?				
Mean ± SD	1.85 ± 0.8	2.14 ± 0.6	2.09 ± 0.6	0.151
MSWS scale total scores categories				
Not at all	6.0 (46.2)	8.0 (12.3)	14 (17.9)	0.009
Moderately	3.0 (23.1)	38 (58.5)	41 (52.6)	
Extremely	4.0 (30.8)	19 (29.2)	23 (29.5)	
Part III: Social Functioning Questionnaire (SFQ): Please look at the statements below and tick the reply that comes closest to how you have been recently (or in the past 2 weeks for studies involving repeated measurement)				
1. I complete my tasks at work and home satisfactorily				
Mean ± SD	1.31 ± 0.9	1.94 ± 0.8	1.83 ± 0.8	0.015
2. I find my tasks at work and at home very stressful				
Mean ± SD	1.46 ± 0.9	2.34 ± 0.7	2.19 ± 0.8	0.001
3. I have no money problems				
Mean ± SD	1.23 ± 1.1	1.17 ± 0.9	1.18 ± 1.0	0.843
4. I have difficulties in getting and keeping close relationships				
Mean ± SD	1.08 ± 1.1	1.25 ± 0.8	1.22 ± 0.8	0.536
5. I have problems in my sex life				
Mean ± SD	1.31 ± 0.9	0.89 ± 0.6	0.96 ± 0.7	0.067
6. I get on well with my family and other relatives				
Mean ± SD	0.62 ± 0.8	1.05 ± 0.8	0.97 ± 0.8	0.115
7. I feel lonely and isolated from other people				
Mean ± SD	1.23 ± 1.0	1.65 ± 1.1	1.58 ± 1.1	0.221
8. I enjoy my spare time				
Mean ± SD	1.08 ± 0.7	1.06 ± 0.7	1.06 ± 0.7	0.946
Social functioning questionnaire scale total scores categories				
Normal social functioning (SFQ < 10)	6.0 (46.2)	26 (40.0)	32 (41.0)	0.762
Significant impairment in social functioning (SFQ ≥ 10)	7.0 (53.8)	39 (60.0)	46 (59.0)	

Categorical variables are presented as frequencies and percentages. Comparisons were performed using the chi-square test or Fisher’s exact test, as appropriate. Continuous variables were compared using the independent-samples *t*-test.

Table 5 presents the results of the multiple linear regression analysis examining the association between fatigue severity and functional status among patients with multiple sclerosis after adjusting for age, body mass index (BMI), onset of MS symptoms, pain severity, and the presence of chronic diseases. The overall regression model was statistically significant (*F* = 11.984, *p* < 0.001), explaining 54.5% of the variance in fatigue severity (*R*^2^ = 0.545; adjusted *R*^2^ = 0.500). The MSWS-12 total score (*β* = 0.371, *p* = 0.010) and IADL total score (*β* = 0.311, *p* = 0.033) were independently associated with fatigue severity, indicating that poorer walking ability and lower instrumental activities of daily living were associated with higher fatigue severity. In contrast, the SFQ total score was not independently associated with fatigue severity after adjustment (*β* = 0.200, *p* = 0.095).

**Table 5:** Multiple linear regression analysis of the association between fatigue severity and functional status among patients with Multiple Sclerosis (*n* = 78).

Variables	B	SE	*β*	*t*	*p* value	95.0% Confidence Interval	
						Lower bound	Upper bound
Constant	5.904	3.818	–	1.546	0.127	−1.711	13.519
IADL scale total scores	6.906	3.173	0.311	2.177	0.033	0.578	13.233
MSWS scale total scores	0.263	0.099	0.371	2.662	0.010	0.066	0.461
SFQ scale total scores	0.200	0.118	0.200	1.692	0.095	−0.036	0.436
Model statistics	*R* = 0.738	*R*^2^ = 0.545	Adjusted *R*^2^ = 0.500	F = 11.984	<0.001	Durbin–Watson = 1.383	

IADL, The Lawton Instrumental Activities of Daily Living Scale; MSWS, Multiple Sclerosis Walking Scale; SFQ, Social Functioning Questionnaire. Dependent variable: Fatigue Severity Scale (FSS) total score. Model adjusted for age, BMI, onset of MS symptoms, severity of pain, and presence of chronic diseases. Significant at *p* < 0.05.

## Discussion

The present study aimed to assess the association between fatigue and functional status among patients with MS. The findings demonstrated that fatigue was highly prevalent among the study participants, with nearly half experiencing severe fatigue and more than half reporting moderate fatigue. Furthermore, fatigue severity was significantly correlated with impaired instrumental activities of daily living, greater walking impairment, and poorer social functioning. However, after adjustment for potential confounders using multiple linear regression, only instrumental activities of daily living and walking impairment remained independently associated with fatigue severity. However, because of the cross-sectional design, these findings should not be interpreted as evidence of a causal relationship.

The stronger association between fatigue and instrumental activities of daily living compared with social functioning may reflect the direct impact of fatigue on activities that require sustained physical and cognitive effort, such as shopping, food preparation, transportation, and medication management. These tasks depend heavily on endurance, mobility, executive functioning, and sustained attention, all of which are commonly affected in multiple sclerosis. In contrast, social functioning is influenced by a broader range of psychological, environmental, and interpersonal factors, including family support, employment status, coping strategies, and emotional well-being. Consequently, the contribution of fatigue to social functioning may be less direct and may diminish after adjustment for other clinical and functional variables. The relationship between fatigue and impaired functional status is likely multifactorial. Central fatigue in multiple sclerosis has been linked to widespread neuroinflammation, demyelination, and disrupted neural connectivity within cortico-striatal and cortico-thalamic pathways, resulting in impaired motor control, reduced cognitive efficiency, and increased perceived effort during daily activities. Cognitive fatigue may further compromise attention, executive functioning, and information processing speed, thereby reducing the ability to perform complex daily tasks independently. These mechanisms may explain why fatigue is particularly associated with instrumental activities of daily living and walking performance.

The current study showed that the majority of participants were female and were in their third and fourth decades of life. This finding is consistent with the well-established epidemiological pattern of MS, which predominantly affects women and typically manifests during early adulthood. Similar demographic distributions have been reported in recent studies conducted in Egypt and internationally, where females represented the majority of MS populations and the mean age ranged between 30 and 40 years (22). The predominance of women in the current study may partly explain the relatively high burden of fatigue and functional limitations observed, as previous research has suggested that women with MS often report greater symptom severity and poorer quality-of-life outcomes than men (23). Regarding fatigue severity, female participants demonstrated higher fatigue scores than males across most fatigue dimensions, although statistically significant differences were observed only for the perception of fatigue as one of the most disabling symptoms. Additionally, severe fatigue was more prevalent among females than males. These findings are consistent with previous studies that reported greater fatigue severity among women with MS (24, 25). Maier et al. (24) reported that female patients frequently experience more severe fatigue symptoms, possibly because of hormonal influences, differences in immune responses, psychological distress, and greater caregiving and household responsibilities. The absence of statistically significant differences in overall fatigue scores between sexes may be attributed to the relatively small number of male participants in the present study.

The present study revealed that although most participants remained independent in certain instrumental activities of daily living, a considerable proportion demonstrated dependence in more physically demanding activities such as shopping, transportation, and food preparation. Furthermore, female participants exhibited lower overall functional independence than males. These findings are consistent with those reported by Weiland et al. (26), who found that fatigue and disease-related limitations were associated with reduced participation in daily activities and diminished functional independence among individuals with MS. The observed reduction in functional independence may be explained by the cumulative effects of fatigue, mobility limitations, pain, and disease progression, all of which interfere with the ability to perform complex daily tasks independently. Walking impairment was another prominent finding in the current study. Participants reported the greatest difficulties in running, stair climbing, balance maintenance, and walking endurance. Female participants also experienced significantly greater need for indoor walking support and were more likely to report moderate or severe walking impairment. These findings agree with previous studies indicating that fatigue is closely associated with gait disturbances, reduced walking speed, impaired balance, and decreased mobility in patients with MS (27, 28). The observed association between fatigue and walking dysfunction may reflect several potential mechanisms, impaired neuromuscular performance, increased energy expenditure during movement, and reduced physical endurance associated with disease-related neurological damage. With regard to social functioning, more than half of the participants demonstrated significant social impairment. Female participants reported greater difficulties completing work and home-related tasks and experienced higher levels of stress associated with these responsibilities. Although fatigue was correlated with poorer social functioning in the present study, social functioning was not independently associated with fatigue severity after adjustment for other clinical and functional variables. This suggests that the relationship between fatigue and social participation may be influenced by additional factors, including mobility limitations, disease characteristics, and functional restrictions (29). Higher fatigue levels may be associated with reduced participation in social activities and may contribute to emotional distress, social withdrawal, and reduced engagement in meaningful life roles.

The most important finding of the present study was that fatigue severity was significantly associated with key domains of functional status, particularly instrumental activities of daily living and walking ability. Although significant correlations were observed between fatigue and social functioning, social functioning did not remain an independent correlate of fatigue severity after controlling for potential confounders. Fatigue severity demonstrated a strong positive association with dependence in instrumental activities of daily living, walking impairment, and social dysfunction. The strongest relationship was observed with instrumental activities of daily living, followed by walking impairment and social functioning. These findings are consistent with previous studies reporting that fatigue is closely associated with disability and functional impairment among individuals with MS. Similar findings were reported by Kratz et al. (30), who demonstrated that higher fatigue levels were associated with reduced physical activity and poorer functional outcomes. Likewise, previous studies have reported that fatigue is associated with mobility limitations, reduced participation in daily activities, and poorer quality of life among individuals with MS (31). Although the observed associations may reflect several biological and behavioral mechanisms, the cross-sectional nature of this study precludes conclusions regarding the direction of these relationships. Fatigue may be related to reduced physical endurance, increased perceived effort during activity, and difficulties sustaining performance over time. However, longitudinal studies are required to clarify the direction of these relationships. Additionally, cognitive fatigue may impair concentration, decision-making, and task completion, thereby affecting both daily functioning and social participation. Consequently, patients with higher fatigue levels also reported greater functional dependence and social impairment in this study. These findings emphasize the importance of early identification and effective management of fatigue as an integral component of comprehensive MS care.

### Strengths and limitations

This study has several strengths, including the comprehensive assessment of fatigue and functional status across multiple domains and the use of standardized assessment instruments. Additionally, the application of multiple linear regression allowed adjustment for important clinical factors, including age, disease onset, pain severity, and chronic diseases, providing a more robust assessment of factors independently associated with fatigue severity. Nevertheless, several limitations should be acknowledged. The cross-sectional design prevents conclusions regarding causal or temporal relationships between fatigue and functional status. Since fatigue and functional outcomes were measured at the same time point, reverse causation cannot be excluded. Furthermore, the relatively small sample size, particularly the limited number of male participants, which may have reduced the statistical power of the analyses and restricted the number of covariates that could be included in the multivariable regression model. Although the regression analyses were adjusted for the principal confounders available in the dataset, other important clinical variables, including disability severity (Expanded Disability Status Scale), depression, anxiety, sleep quality, cognitive impairment, and disease-modifying therapy, were not assessed. Therefore, residual confounding cannot be excluded, and the findings should be interpreted with caution. Additionally, information regarding disease-modifying therapy (DMT) use was not collected in the present study. Because DMTs may influence fatigue severity, disease activity, and functional outcomes, the absence of these data may have resulted in residual confounding. Moreover, both the Fatigue Severity Scale and the Multiple Sclerosis Walking Scale were administered using modified three-point response formats rather than their original validated scoring systems. Although these adaptations facilitated questionnaire administration, the resulting fatigue and walking impairment categories may not be directly comparable with findings from studies using the original validated versions of these instruments. Future studies should incorporate treatment-related variables to better evaluate their potential influence on the relationship between fatigue and functional status.

## Conclusion

The present study demonstrated that fatigue is highly prevalent among patients with MS and is significantly associated with functional limitations. After adjustment for potential confounding factors, greater walking impairment and poorer instrumental activities of daily living remained independently associated with higher fatigue severity, whereas social functioning did not demonstrate an independent association. These findings highlight the importance of routine fatigue assessment and comprehensive functional evaluation, particularly regarding mobility and daily living activities, to support individualized nursing and rehabilitation care for patients with MS.

## Data Availability

The raw data supporting the conclusions of this article will be made available by the authors, without undue reservation.
